# Supplementary material for: DAZAP2 functions as a pan-coronavirus restriction factor by inhibiting viral entry and genomic replication
Source: mBio. 2025 Aug 20;16(9):e00385-25. doi: 10.1128/mbio.00385-25 (PMC12421815; doi:10.1128/mbio.00385-25)
Supplement: Supplemental figures — Fig. S1-S6. [file mbio.00385-25-s0001.pdf]

SUPPLEMENTARY FIGURES LEGENDS

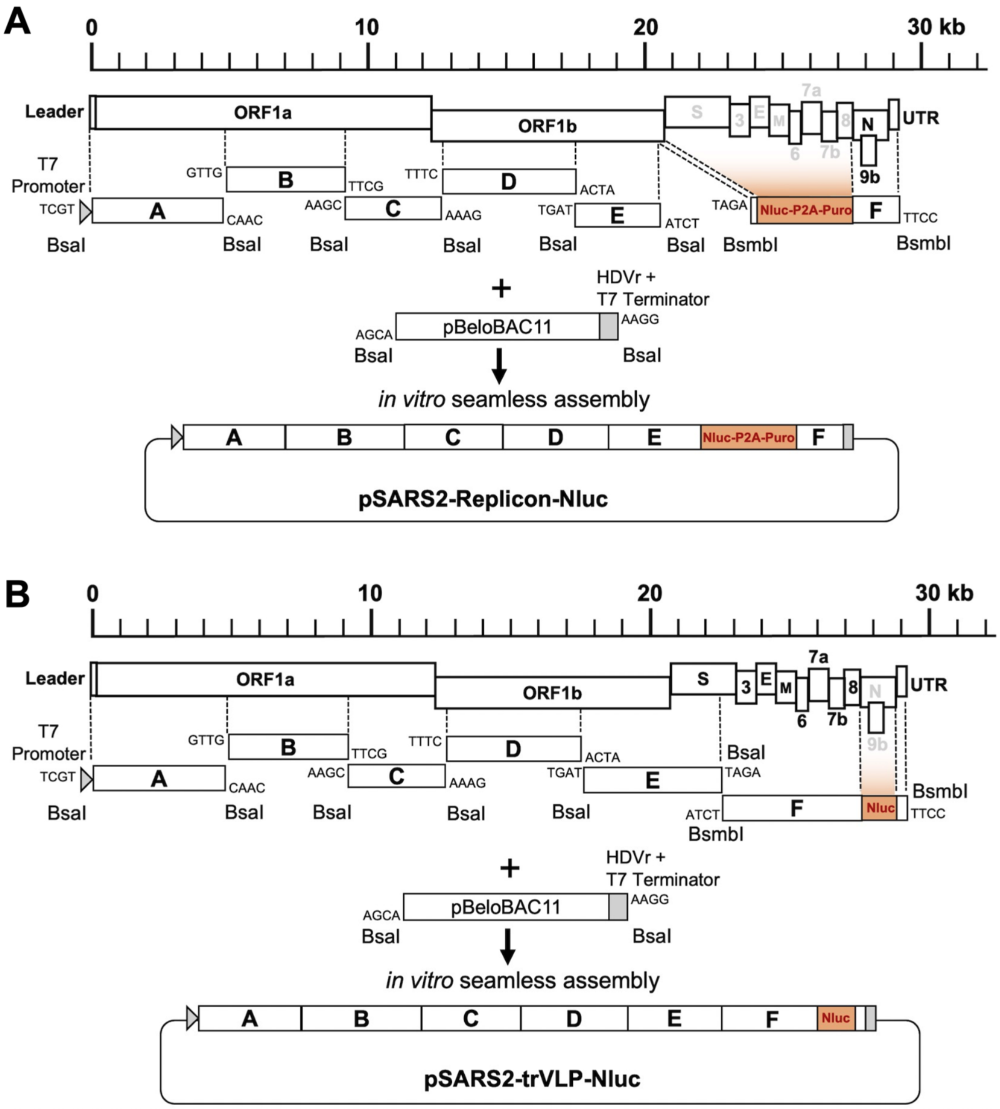

Supplementary Figure 1. Generation of SARS-CoV-2 replicon system and trVLP-Nluc particles. A. Schematic of the construction of SARSC-CoV-2 replicon system. The genome of SARS-CoV-2 was divided into 6 fragments, amplified, and cloned into pSMART vectors. The genes from spike to ORF8 were replaced by a NanoLuc luciferase-P2A-puromycin cassette. Viral fragments were cleaved from the vectors and assembled with the linearized pBeleBAC11 vector *in vitro* using type IIS restriction enzymes. The transcription of replicon RNA was initiated by a T7 promoter and terminated

by a T7 terminator. The HDVr sequences were added after the poly-A tail to obtain the correct viral RNA. **B.** Schematic of the generation of trVLP-Nluc particles. Based on the replicon system constructed above, the spike to ORF8 genes were maintained but the N gene was replaced by NanoLuc luciferase. The trVLP-Nluc particles were packaged in Vero E6 cells expressing the N gene, and could only replicate for a single round in cells without the expression of N protein.

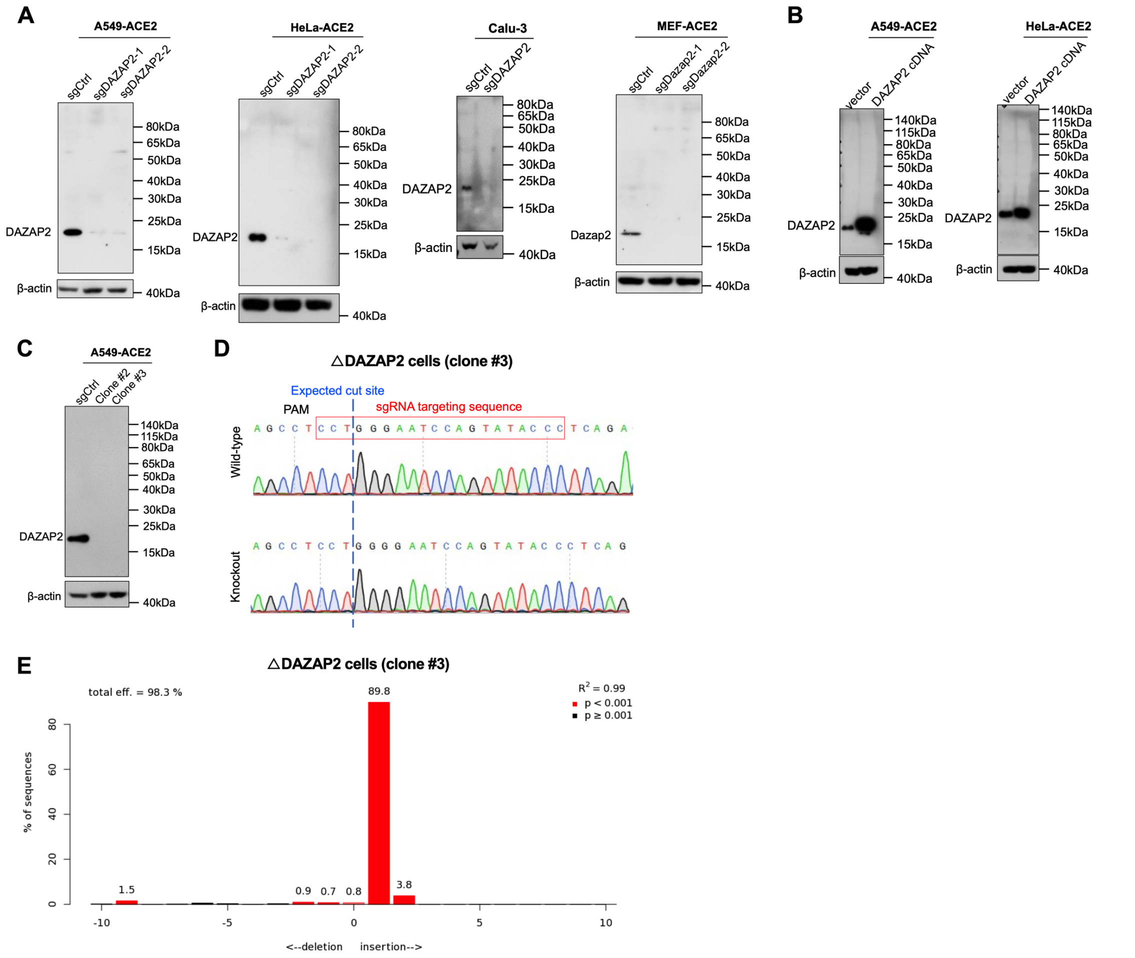

Supplementary Figure 2. Verification of DAZAP2 expression in cDNA-overexpressing, sgRNA-edited bulk or clonal cells. A. Knockout efficiency of *DAZAP2* in A549-ACE2, HeLa-ACE2, Calu-3, and MEF-ACE2 cells. Two sgRNAs or one

representative sgRNA were used, and gene-edited bulk cells were subjected to western blotting. **B.** Overexpression of DAZAP2 in A549-ACE2 or HeLa-ACE2 cells. Cells were transduced with lentivirus bearing the human DAZAP2 cDNA, and subjected to western blotting. **C.** Western blotting to verify the *DAZAP2*-knockout clones #2 and #3 of A549-ACE2**.** Clone #3 (ΔDAZAP2) was used in this study. **D.** The sequence traces of the *DAZAP2* gene locus of WT and clonal cells. The sgRNA target site is indicated. **E.** The ICE analysis of the ΔDAZAP2 clonal cell line.

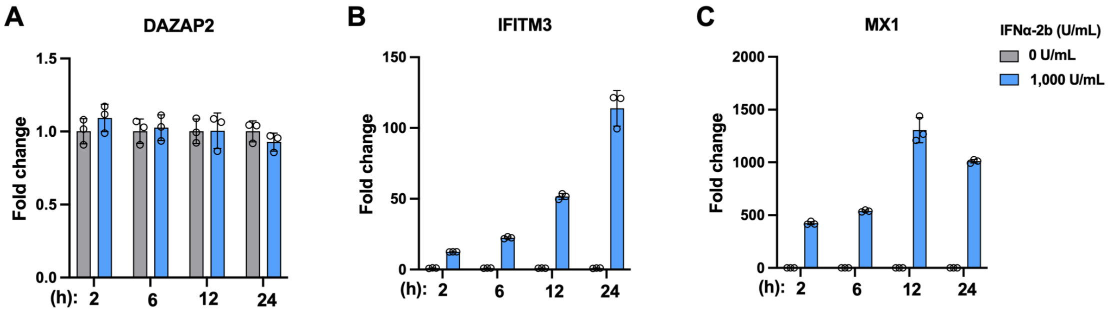

Supplementary Figure 3. DAZAP2 is not an interferon-stimulated gene (ISG). A-C. A549-ACE2 cells were treated with 0 or 1,000 U/ml of IFNα-2b for various time points, and cellular RNA was extracted for detection of *DAZAP2* (**A**), *IFITM3* (**C**), or *MX1* (**C**) by qRT-PCR. The experiment was performed in triplicate and data were normalized to the treatment with 0 U/ml of IFNα-2b at each time point.

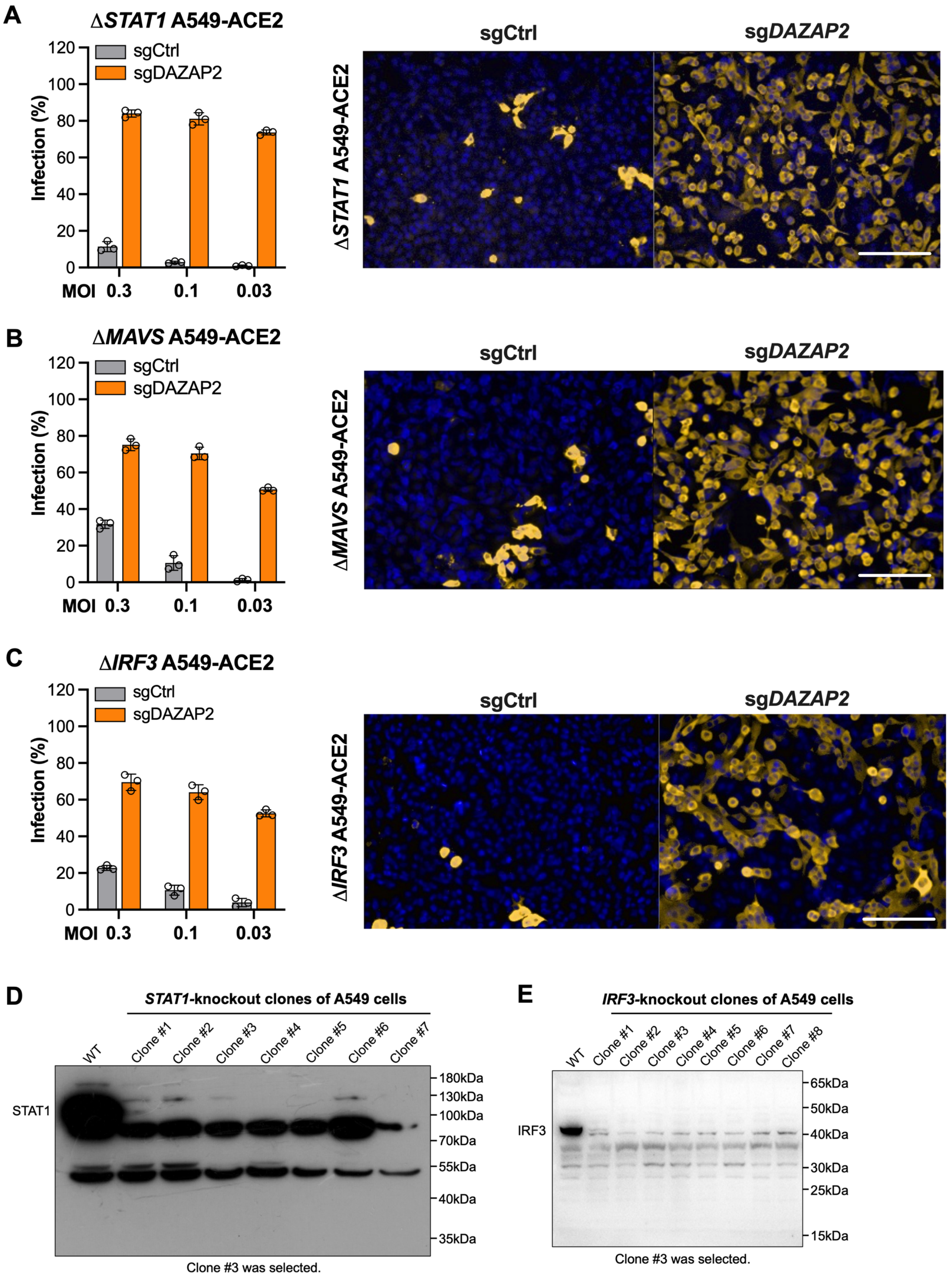

Supplementary Figure 4. The antiviral effect of DAZAP2 in innate immune gene-knockout cells. A-C. The antiviral effect of DAZAP2 on SARS-CoV-2 infection in *STAT1*-, *MAVS*-, or *IRF3*-knockout A549-ACE2 cells. Cells were infected with different

MOIs and the percentage of N positive cells were analyzed (left panels of A to C). The representative fluorescence images from high-content analysis in cells were shown on right panels of A to C. **D-E.** Verification of *STAT1*- or *IRF3*-knockout efficiency in clonal cell lines by Western blotting. The clone #3 for *STAT1*- or *IRF3*-knockout cells was selected for use.

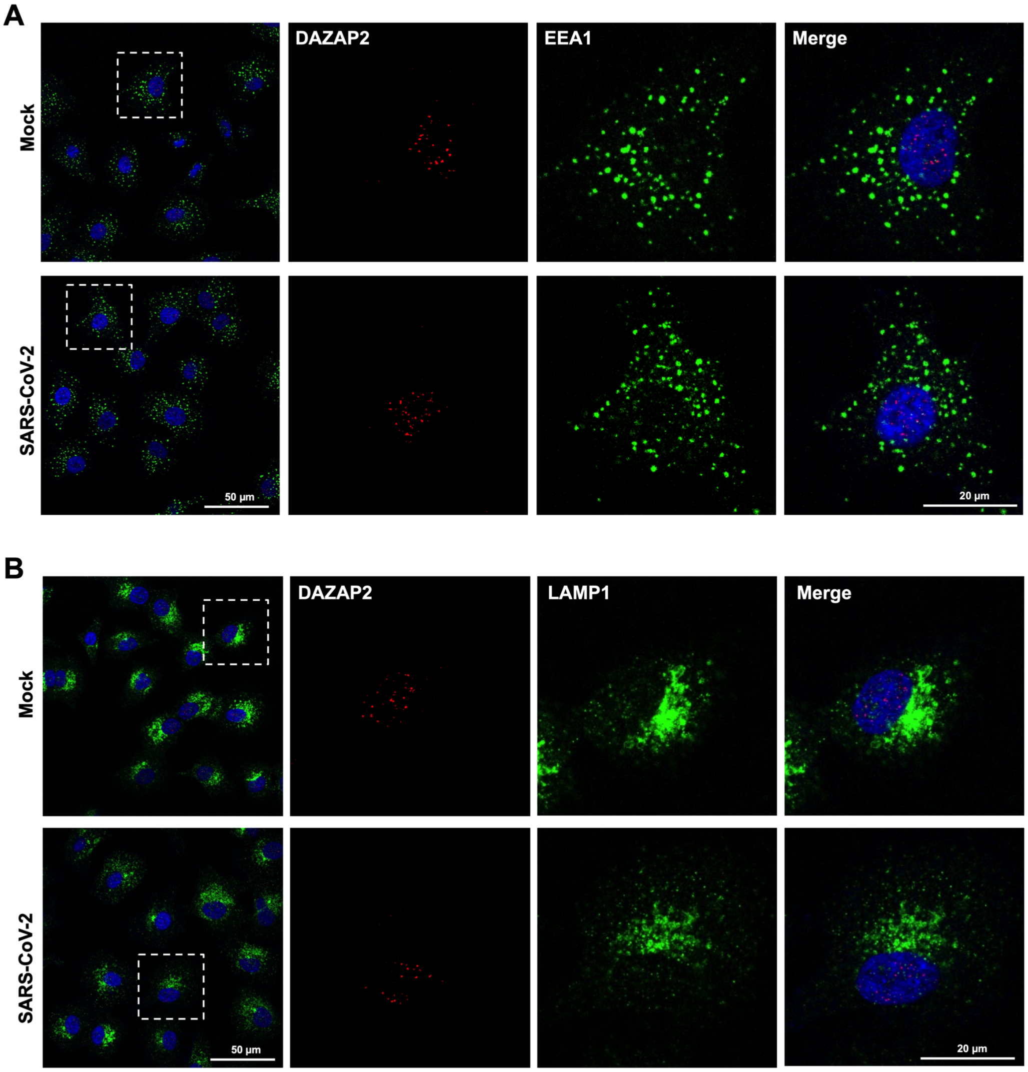

Supplementary Figure 5. Localization study of DAZAP2 by confocal microscopy. A-B. The A549-ACE2 cells were infected with SARS-CoV-2, then fixed and

stained with anti-DAZAP2 (A and B), anti-EEA1 (A), or anti-LAMP1 (B) antibody. The representative confocal images were shown. Scale bar, 50 or 20 μm.

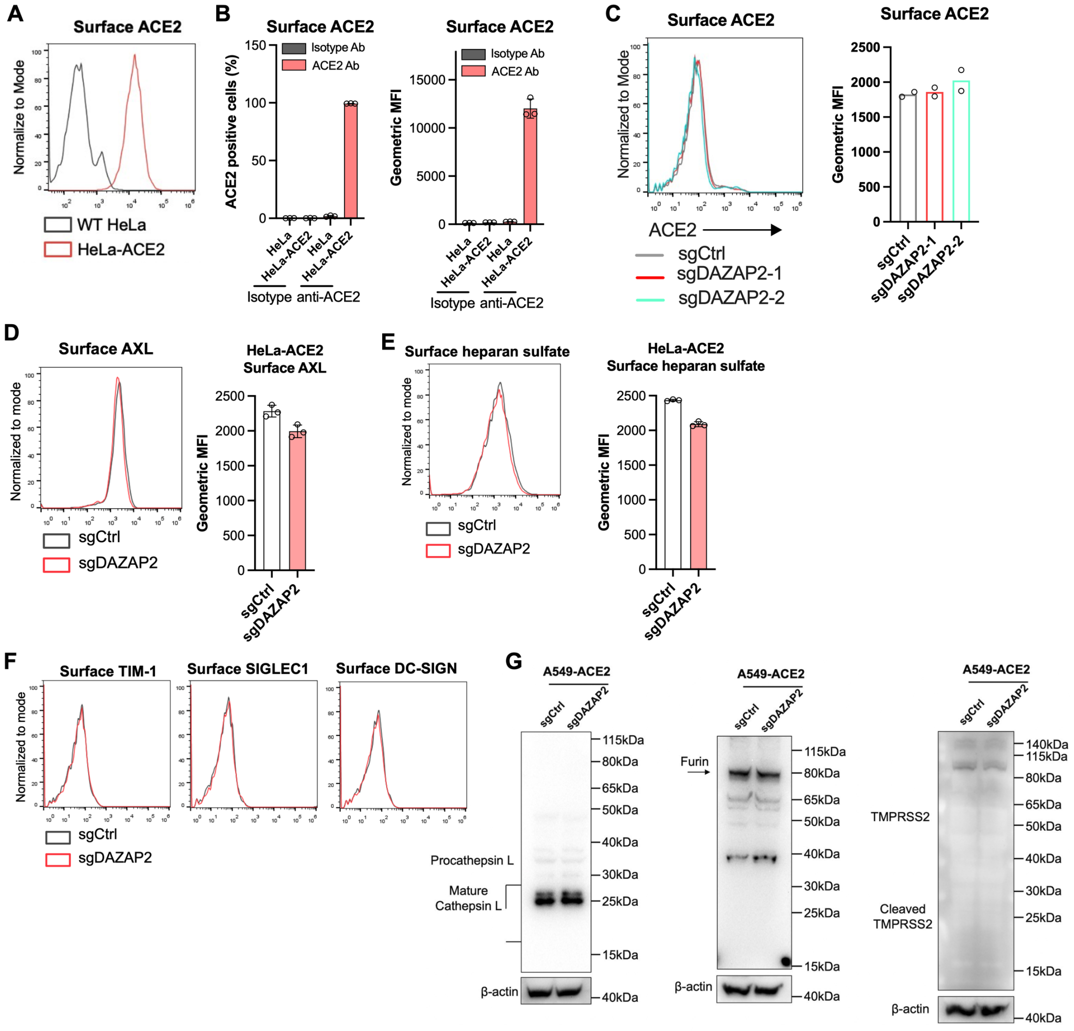

Supplementary Figure 6. The expression of SARS-CoV-2 entry-related host factors. A. Flow cytometry analysis of surface expression of ACE2 in WT or ACE2-overexpressing HeLa cells stained with anti-ACE2 antibody. **B.** Flow cytometry analysis of surface expression of ACE2 in WT or ACE2-overexpressing HeLa cells stained with isotype

or anti-ACE2 antibody. The percentage of ACE2 positive cells and geometric mean fluorescence intensity (MFI) were analyzed. **C-E.** Flow cytometry analysis of surface expression of ACE2, AXL, or heparan sulfate in A549-ACE2 (C) or HeLa-ACE2 (D and E) cells edited with control or *DAZAP2* sgRNA. The percentage of positive cells and geometric mean fluorescence intensity (MFI) were analyzed. **F.** Flow cytometry analysis of surface expression of TIM-1, SIGLEC1, or DC-SIGN in A549-ACE2 cells edited with control or *DAZAP2* sgRNA. **G.** Western blotting analysis of CTSL, Furin, or TMPRSS2 in A549-ACE2 cells edited with control or *DAZAP2* sgRNA.

SUPPLEMENTARY TABLE LEGENDS

Supplementary Table 1. List of genes and scores after MaGeck analysis (see Excel file). Data was obtained by sequencing the sgRNAs from uninfected or sorted cells.

Supplementary Table 2. sgRNA sequences of genes selected for validation and other editing experiments (see Excel file).

Supplementary Table 3. List of primers and probes used for qRT-PCR experiments (see Excel file).
